# Abdominal and Back Pain Initially Suspected to Be Transient Intestinal Ischemia in a Case of Streptococcus dysgalactiae subsp. equisimilis Bacteremia

**DOI:** 10.7759/cureus.101031

**Published:** 2026-01-07

**Authors:** Daichi Yokobori, Taichi Fujimori, Ryuichi Ohta, Chiaki Sano

**Affiliations:** 1 Internal Medicine, Masuda Red Cross Hospital, Masuda, JPN; 2 Community Care, Unnan City Hospital, Unnan, JPN; 3 Community Medicine Management, Shimane University Faculty of Medicine, Izumo, JPN

**Keywords:** atypical presentation, back pain, bacteremia, cellulitis, elderly, streptococcus dysgalactiae subsp. equisimilis

## Abstract

Streptococcus dysgalactiae subsp. equisimilis (SDSE) has been increasingly recognized as a causative pathogen of skin and soft tissue infections and bacteremia, particularly in elderly individuals or those with underlying comorbidities. It typically presents with fever and localized pain associated with cellulitis; however, it may occasionally manifest with nonspecific symptoms, leading to diagnostic delay.

We report the case of an 87-year-old man who presented with abdominal and lower back pain, initially suspected to have intestinal ischemia based on contrast-enhanced computed tomography findings and elevated serum lactate levels, but was later diagnosed with SDSE bacteremia following positive blood cultures and subsequent identification of cellulitis between the right third and fourth toes.

After initiation of antimicrobial therapy with ampicillin/sulbactam and intravenous fluids, his fever and inflammatory response improved. Based on the clinical course, it was suggested that bacteremia-related circulatory disturbances and transient intestinal ischemia might have contributed to the nonspecific abdominal and back pain.

This case highlights the importance of recognizing SDSE bacteremia as a potential etiology of atypical initial symptoms - such as abdominal or back pain - even before the development of overt skin lesions.

## Introduction

Streptococcus dysgalactiae subsp. equisimilis (SDSE) is classified as a group C or group G *β-hemolytic streptococcus *and has attracted increasing attention as a causative pathogen of invasive infections in elderly individuals. Reports indicate a rising incidence of SDSE bacteremia over recent decades, particularly in Northern European countries and Japan, with an estimated incidence of approximately 17 cases per 100,000 population [1]. The median age at onset is around 70 years, and most affected patients are elderly or have underlying conditions such as diabetes mellitus, cardiovascular disease, malignancy, or immunosuppression [2,3]. Bacteremia secondary to skin and soft tissue infections, such as cellulitis, is the most common presentation, accounting for approximately 70% of all cases [1,4]. Although the infectious source remains unidentified in approximately 20% of patients, some case-based reports suggest that SDSE bacteremia can precede the appearance of overt skin lesions, presenting with nonspecific symptoms such as fever, fatigue, abdominal pain, or back pain, and may initially be misdiagnosed as a gastrointestinal or musculoskeletal disorder [3-5]. Here, we report a case of an elderly man who presented with abdominal and lower back pain, initially suspected to have intestinal ischemia, but was ultimately diagnosed with SDSE bacteremia associated with cellulitis. A brief literature review is also provided.

## Case presentation

An 87-year-old man presented to our hospital on day 0 with diarrhea, vomiting, abdominal pain, and excruciating lower back pain. He lived alone and was independent in activities of daily living. He consumed alcohol daily. On the morning of admission, he had breakfast and subsequently took a nap at his home. Upon awakening, he experienced diarrhea, followed in sequence by vomiting, abdominal pain, and intense lower back pain described as excruciating. This episode was also accompanied by fecal incontinence. His primary care doctor visited him emergently at home and advised hospitalization at our hospital, after which he was transported to our emergency department by ambulance. His medical history was notable primarily for advanced age, with multiple chronic comorbidities. These included atrial fibrillation, cerebellar infarction, chronic enteritis, neurosis, gallbladder polyp, a history of malignant lymphoma treated with transplantation, bronchial asthma, lumbar spinal canal stenosis, and glaucoma.

His regular medications included nizatidine (75 mg twice daily), lemborexant (2.5 mg at bedtime), acetaminophen (200 mg twice after each meal), dapagliflozin (5 mg once daily), febuxostat (10 mg once daily), eplerenone (25 mg once daily), edoxaban (30 mg once daily), bisoprolol (2.5 mg, half tablet once daily), and tafluprost/timolol ophthalmic solution once daily in both eyes.

On arrival, he was alert and oriented. His temperature was 37.4°C, and his blood pressure was 99/55 mmHg, which was slightly lower than his usual outpatient baseline. His heart rate was 75 beats per minute with an irregular rhythm, respiratory rate 12 breaths/min, and oxygen saturation 95% on room air. Lung sounds were clear bilaterally, and cardiac auscultation revealed a third heart sound. The abdomen was flat and soft but tender in the left lateral region, with abdominal percussion tenderness, and left costovertebral angle tenderness. No rash or leg edema was observed, and dorsalis pedis pulses were palpable bilaterally.

At admission, the back pain was extremely severe (Numerical Rating Scale > 10) and qualitatively different from his chronic pain. The pain occurred paroxysmally and resolved spontaneously within tens of minutes. As-needed acetaminophen (500 mg) was administered during these episodes; however, the short duration and self-limited nature of the pain made it difficult to determine a specific aggravating or relieving factor. This clinical pattern raised the possibility of a transient underlying process.

Laboratory findings and imaging

Laboratory findings on day 0 demonstrated marked leukocytosis with neutrophil predominance, mild anemia, and mild thrombocytopenia, representing a slight decrease from the patient’s baseline platelet count (approximately 12.7 × 10^4^/µL). Blood glucose levels were elevated, while HbA1c remained within the normal range. Blood urea nitrogen and creatinine were mildly increased, indicating mild renal impairment. Serum lactate was also elevated, suggesting impaired perfusion. Liver enzymes, creatine kinase, and electrolytes were within normal limits.

Urinalysis revealed strong glucose positivity, likely attributable to the patient’s use of a sodium-glucose cotransporter 2 inhibitor (dapagliflozin). Bacteria were observed on urine microscopy, and Gram staining demonstrated gram-positive cocci. Urine culture grew Aerococcus sanguinicola; however, the patient had no urinary symptoms, and this finding was considered incidental and unrelated to the confirmed Streptococcus dysgalactiae subsp. equisimilis bacteremia. Detailed laboratory values are summarized in Table 1.

**Table 1:** Initial laboratory data of the patient Laboratory findings on admission. Reference ranges are shown in the rightmost column. Urinalysis results are also included.

Parameter	Level	Reference
White blood cells	18.40	3.5–9.8 × 10^3^/μL
Neutrophils	90.1	44.0–72.0%
Lymphocytes	2.8	18.0–59.0%
Hemoglobin	10.8	11.3–15.2 g/dL
Hematocrit	32.8	33.4–44.9%
Mean corpuscular volume	98.6	79.0–100.0 fL
Platelets	11.2	13.0–36.9 × 10^4^/μL
Total protein	6.6	6.5–8.3 g/dL
Albumin	3.2	3.8–5.3 g/dL
Total bilirubin	0.8	0.2–1.2 mg/dL
Aspartate aminotransferase	15	8–38 IU/L
Alanine aminotransferase	10	4–43 IU/L
Lactate dehydrogenase	179	121–245 U/L
Blood urea nitrogen	33.4	8–20 mg/dL
Creatinine	1.56	0.40–1.10 mg/dL
Serum Na	137	135–150 mEq/L
Serum K	4.5	3.5–5.3 mEq/L
Serum Cl	105	98–110 mEq/L
Ferritin	92.4	14.4–303.7 ng/mL
Blood glucose	270	70−110 mg/dL
HbA1c	6.1	5.0−6.2%
Serum lactate	3.1	0.5−1.6 mmol/L
Urine test		
Leukocyte	(-)	(-)
Protein	(-)	(-)
Glucose	(4+)	(-)
Blood	(+-)	(-)
Bacteria	(2+)	(-)

**Figure 1:**
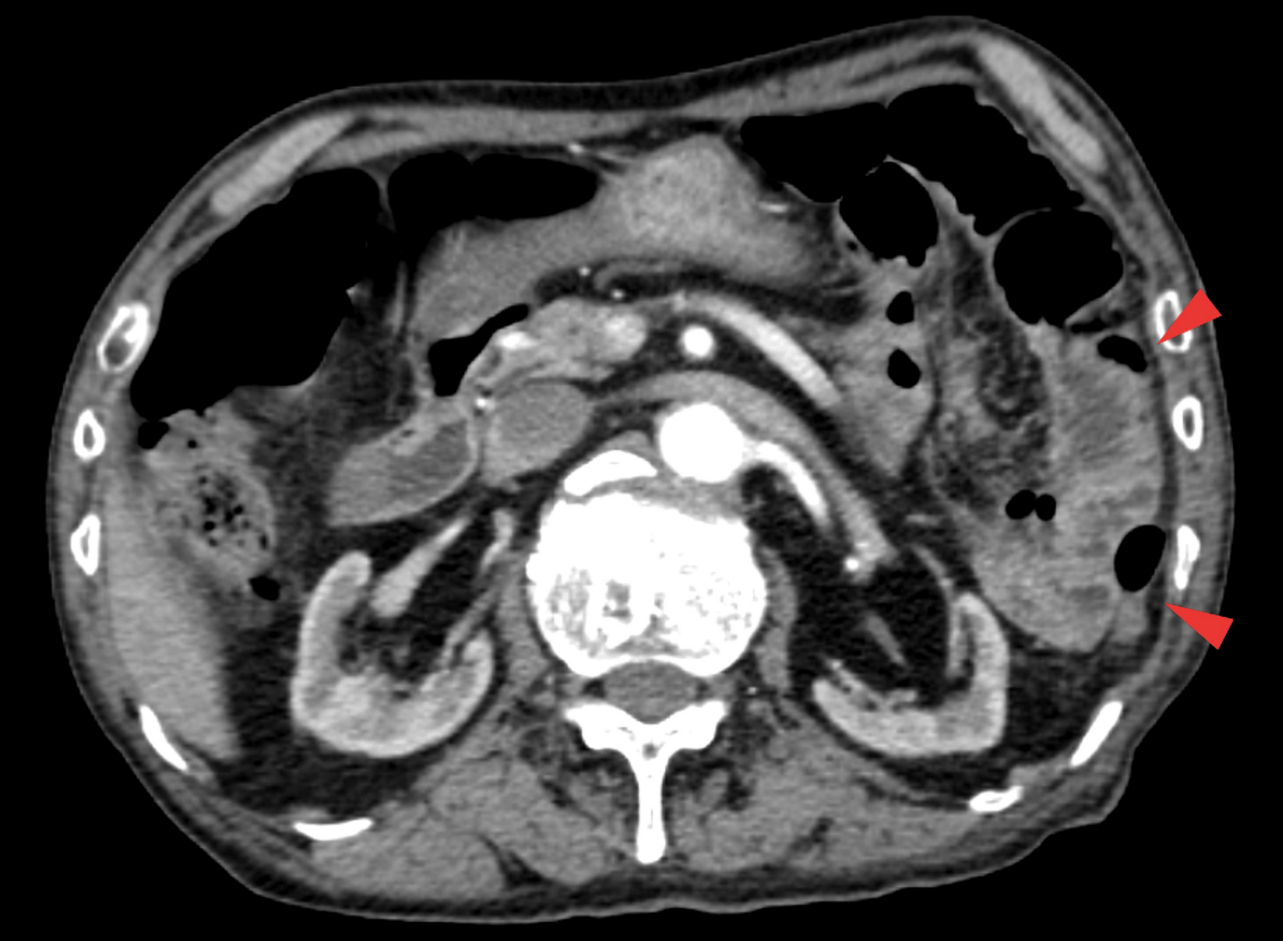
Contrast-enhanced abdominal CT (axial view) Thickened wall of the left colon and surrounding fat stranding (arrowheads) are evident.

**Figure 2:**
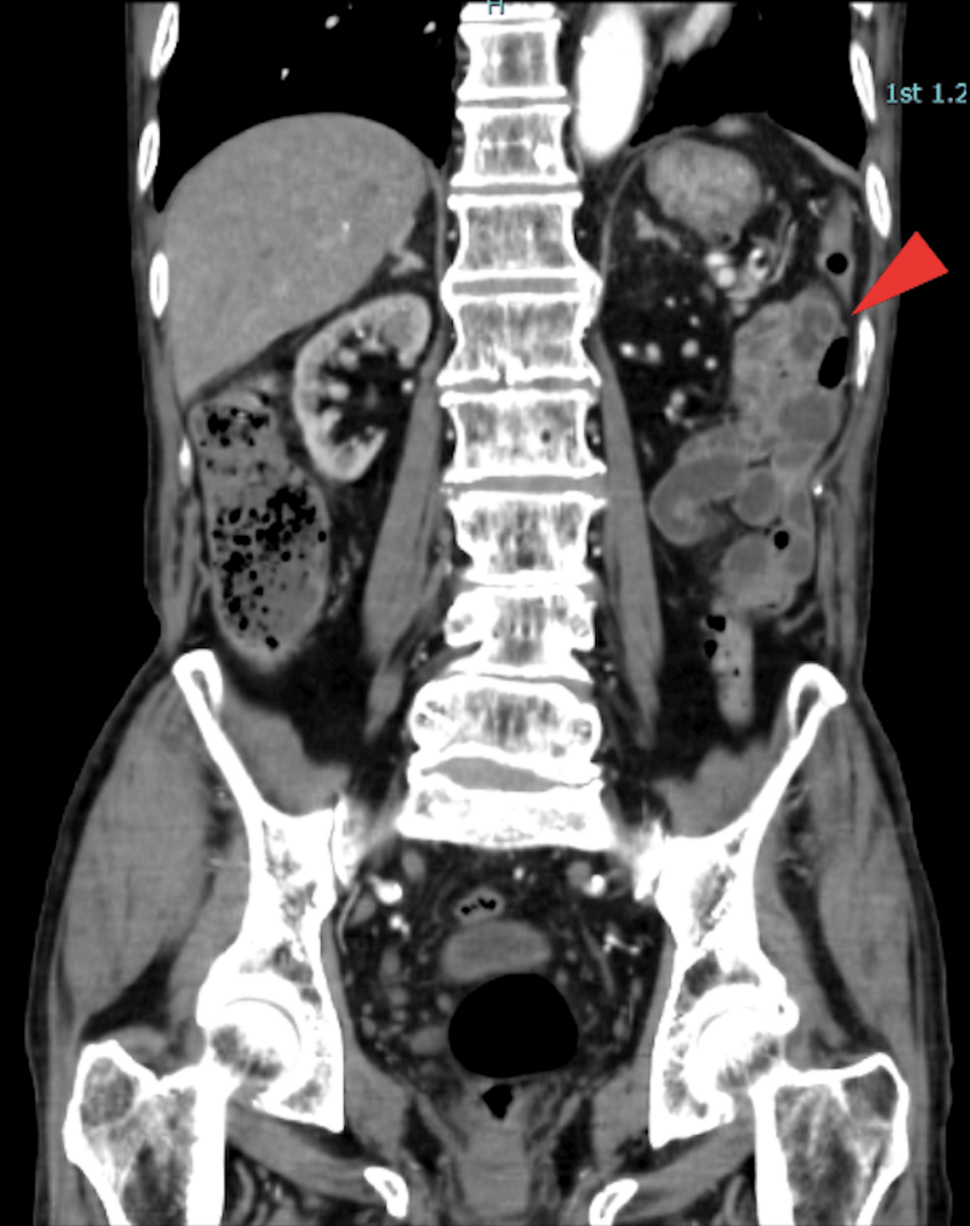
Contrast-enhanced abdominal CT (coronal view) Segmental thickening of the left colon wall with increased perienteric fat density (arrowhead) is observed.

On day 0, contrast-enhanced CT (Computed Tomography) revealed thickening of the left intestinal wall and increased perienteric fat density, findings clinically considered suggestive of possible intestinal ischemia (Figures 1, 2). No thrombus, embolism, dissection, abscess, or perforation was detected. Marked atherosclerosis suggested transient intestinal ischemia secondary to hemodynamic alterations. Follow-up imaging was not performed because the patient’s abdominal symptoms and serum lactate levels subsequently improved.

Clinical course

The patient was initially managed with intravenous fluids, but abdominal pain recurred without improvement in lactate levels. On day 1, the lactate level normalized; however, low-grade fever persisted. Two sets of blood cultures yielded Gram-positive cocci in chains (GPC in chains). Empirical ampicillin/sulbactam (ABPC/SBT) 3 g Intravenous (IV) twice daily was started, considering possible pyogenic spondylitis.

**Figure 3:**
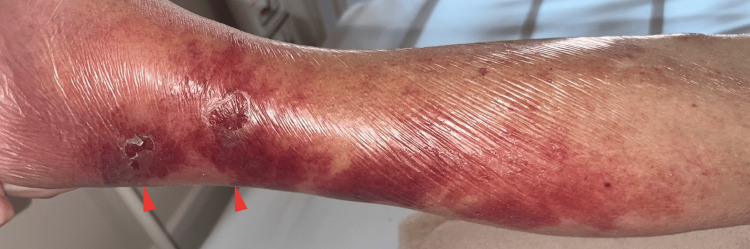
Clinical appearance of cellulitis on the right lower leg Clinical appearance of the right lower leg showing diffuse erythema and swelling with superficial erosions and desquamation, consistent with cellulitis secondary to *Streptococcus dysgalactiae* subsp. *equisimilis* infection. The affected area was tender to palpation, corresponding to the erythematous and swollen region.

By hospital day 4, the fever persisted. Magnetic resonance imaging excluded spondylitis, but erythema and swelling developed in the right lower limb (Figure 3), suggesting cellulitis; therapy was switched to vancomycin (VCM) 0.5 g/day. Subsequently, Streptococcus dysgalactiae subsp. equisimilis was identified using an automated microbiological identification system, and treatment was de-escalated to ampicillin (ABPC) 1 g IV three times daily based on susceptibility testing.

Dermatology consultation revealed a fissure between the third and fourth toes of the right foot, identified as the probable portal of entry. Urine culture also revealed Gram-positive cocci, suggesting possible concordance with the bloodstream organism. Follow-up blood cultures turned negative, confirming bacteremia control. Antibiotic therapy was continued for 14 days after culture clearance. Fever resolved, abdominal and back pain gradually subsided, inflammatory markers normalized, local findings improved, and he was discharged in good condition.

## Discussion

This case represents a rare instance of SDSE bacteremia that initially presented with abdominal and lower back pain, later revealing cellulitis as the primary focus. In our patient, these symptoms occurred abruptly after breakfast and were accompanied by vomiting and fecal incontinence, reflecting a transient intestinal ischemia-like presentation prior to the appearance of skin lesions. Such a sequence is extremely atypical for SDSE infection. This case highlights two important aspects: first, that SDSE bacteremia can manifest initially with severe abdominal or back pain mimicking an intra-abdominal disorder; and second, that clinicians should consider infectious etiologies even in patients presenting with unexplained pain or fever before localizing signs become evident.

SDSE bacteremia typically affects elderly individuals and often manifests as cellulitis, most commonly originating from chronic skin barrier disruptions such as toe web fissures or tinea pedis [3,4]. Although our patient shared this demographic profile - an elderly man with a toe web fissure that likely served as the portal of entry - the clinical course was distinctive in that systemic symptoms, including abdominal and back pain, appeared before any cutaneous findings. Notably, cellulitis of the right lower limb became apparent only on hospital day 4, several days after the onset of bacteremia and systemic inflammatory symptoms. This temporal gap suggests that transient bacteremia and the resulting systemic inflammatory response may have preceded, and possibly precipitated, the subsequent localized infection. In other words, the skin lesion may represent a later, localized expression of an already established systemic process, rather than the initial infectious focus. Such a presentation underscores the pathophysiologic diversity of SDSE infection, in which the clinical phenotype can progress from systemic to localized manifestations over time.

Abdominal and back pain in this patient may have resulted from systemic inflammatory responses and hemodynamic alterations triggered by SDSE bacteremia. Ogura et al. demonstrated in a diabetic mouse model that invasive SDSE infection induces marked cytokine release and endothelial injury, leading to impaired microcirculation [6]. Such mechanisms could explain the transient intestinal ischemia-like presentation in our patient, as evidenced by elevated serum lactate and intestinal wall thickening on computed tomography. Furthermore, microcirculatory disturbance in the paraspinal soft tissues may have contributed to his severe lower back pain. In elderly individuals, aging-associated endothelial dysfunction and arterial stiffness can increase vulnerability to ischemic pain during transient inflammatory or circulatory stress, which may have intensified the symptoms observed in this case [7].

Reports of SDSE bacteremia initially presenting with abdominal or back pain are exceedingly rare. Although nonspecific systemic symptoms preceding cutaneous findings have been documented in some patients, transient ischemia-like presentations involving the intestine or paraspinal region are particularly uncommon [3,5]. In the present case, such symptoms likely reflected a reversible microcirculatory disturbance caused by systemic inflammation during the early phase of bacteremia, even in the absence of overt hypotension. Previous studies have shown that microvascular flow abnormalities can occur despite preserved systemic hemodynamics in patients with early sepsis, and that inflammatory endothelial activation can further disrupt capillary perfusion and tissue oxygen delivery [8,9]. These pathophysiologic insights support the possibility that similar subclinical circulatory alterations contributed to the ischemia-like symptoms observed here. Collectively, these observations broaden the understanding of SDSE bacteremia, suggesting that its pathophysiologic spectrum may extend beyond the well-recognized cellulitis-associated manifestations.

From a diagnostic standpoint, this case underscores that abdominal or back pain in elderly patients may occasionally indicate systemic infection rather than primary gastrointestinal or musculoskeletal disease. When evaluating fever or bacteremia of unknown origin, clinicians should carefully inspect the feet and interdigital spaces, as small fissures or tinea pedis can serve as subtle portals of entry for SDSE. In our patient, a fissure between the right third and fourth toes was identified as the probable source, emphasizing the value of a meticulous physical examination even when the initial presentation appears unrelated to the skin.

From a practical standpoint, obtaining adequate blood cultures is crucial - preferably three sets to maximize sensitivity - and repeating cultures within 48 hours after antimicrobial initiation, as recommended for Staphylococcus aureus bacteremia, may also be valuable for confirming bacterial clearance in SDSE infection. Although specific guidelines for SDSE are lacking, adherence to general principles of bacteremia management can help ensure adequate treatment and timely verification of cure.

## Conclusions

This report describes a rare case of SDSE bacteremia originating from cellulitis in the right toe web space, presenting with abdominal and back pain clinically suggestive of transient intestinal ischemia, although definitive ischemic findings were not confirmed on imaging. In this case, the patient’s initial presentation with abdominal and back pain required differentiation from vascular and other acute abdominal diseases.

In SDSE bacteremia, skin lesions may appear later in the disease course. Therefore, clinicians should suspect infection early, even when symptoms are nonspecific. Particularly in elderly patients, bacteremia and skin infections may present atypically; prompt blood culture collection and thorough source identification are vital to prevent diagnostic delay and disease progression.
